# Challenges and strategies in the administration of a population based mortality follow-back survey design

**DOI:** 10.1186/1472-684X-12-28

**Published:** 2013-08-06

**Authors:** Beverley Lawson, Kristine Van Aarsen, Frederick Burge

**Affiliations:** 1 Department of Family Medicine, Dalhousie University, 5909 Veterans Memorial Lane, Abbie J. Lane Building, 8th Fl, Halifax, NS B3H 2E2, Canada

## Abstract

Population-based mortality follow-back survey designs have been used to collect information concerning end-of-life care from bereaved family members in several countries. In Canada, this design was recently employed to gather population-based information about the end-of-life care experience among adults in Nova Scotia as perceived by the decedent's family. In this article we describe challenges that emerged during the implementation of the study design and discuss resolutions strategies to help overcome them. Challenges encountered included the inability to directly contact potential participants, difficulties ascertaining eligibility, mailing strategy complications and the overall effect of these issues on response rate and subsequent sample size. Although not all challenges were amenable to resolution, strategies implemented proved beneficial to the overall process and resulted in surpassing the targeted sample size. The inability to directly contact potential participants is an increasing reality and limitations associated with this process best acknowledged during study development. Future studies should also consider addressing participant concerns pertaining to their eligibility and use of a more cost effective mailing strategy.

## Findings

### Introduction

Population-based mortality follow-back designs used to survey a cohort of decedents’ next-of-kin or informal caregivers about end-of-life care (EOLC) have been employed in the UK, the US and Italy [1-7]. This approach permits representative sampling of a population of decedents and helps address several sources of bias encountered in prospective designs such as the identification of people who are at end of life in a specific time period, the recruitment of both recipients and non-recipients of services and the non-participation, withdrawal or ethical exclusion of those too ill to participate [2,8,9]. Follow-back studies are viewed as an essential strategy in describing the events around death [10-12]. Such studies, Teno argues, are among the “multiple methods (or strategies), either combined or in sequence, needed to examine a complex, multidimensional phenomenon such as end-of-life care” [8]. Population-based mortality follow-back surveys have efficiently collected data from bereaved family members (informants) on a range of variables that are not available in administrative data, thus providing population-based estimates on EOLC that otherwise would be unattainable [8]. Although some Canadian studies have used after-death methods to interview or survey bereaved informants or family members, these have focused on decedents who received specific end-of-life services and/or were registered within special programs such as those receiving specialized palliative care services and hence, are not population-based [13-18].

In Canada, a population-based mortality follow-back design was recently employed to gather information about the EOLC experience among adults in Nova Scotia as perceived by the decedent’s family. In this article we describe challenges that emerged during the process of implementing the study design and discuss resolutions strategies to help overcome these issues.

### Background about study participants and the process in brief

Bereaved family member participants for the study were identified using the ‘informant’ (also termed ‘next-of-kin’) field on the death certificates of all who died in the Eastern Canadian province of Nova Scotia (population 950,000) over a two year period. The informant is the person providing information about the decedent at time of death and is usually a family member or someone close to them. Initially a maximal population [19] of potentially eligible deaths was identified. This involved the exclusion of records of decedents where cause of death ICD codes were associated with an external cause or medical and surgical complications (such as pregnancy, childbirth, accidents, unintentional injury, motor vehicle accidents, intentional self-harm, assault, legal intervention, events of undetermined intent, operations of war and their sequelae). Additional exclusions at this stage included death certificates of decedents less than 18 years of age, those with unconfirmed cause of death and missing or incomplete ‘informant’ information. We also desired to exclude decedents who had died suddenly or unexpectedly from causes not noted above and situations where the informant identified was not a family member or close to the decedent and/or familiar with their EOLC. These exclusions were only able to be identified if the informant contacted the researchers directly to inform them of the circumstances surrounding death and are addressed as challenges*.* In total, 5848 death records were identified by Nova Scotia Vital Statistics as potentially eligible over the study period.

Sample size calculations based on past mortality statistics and analyses plans suggested a total sample size of 1200 interviews was required [20]. Bereaved family members of potentially eligible death certificates were identified in ‘waves’, every four months, over a 24 month period for a planned total of six waves. Death certificates identified during each wave were limited to those occurring between four to seven months prior to each wave selection date. This strategy was employed to ensure contact with the bereaved was not immediate but remained relatively consistent. It was also used to promote completion of each survey interview within ten months of the decedent’s date of death, maximize response [21] and to facilitate recall by providing a consistent yet relatively short and acceptable period of time [22,23].

By mail, each identified bereaved family member was sent an invitation package inviting their participation and study information. Recipients were asked to use a response option of their choice (mail, telephone, email) to indicate whether or not they wished to participate or required additional information. Those who agreed were asked to provide their telephone contact information. If they did not feel they were the most informed about the decedent’s EOLC, suggestions for an alternate person to whom the invitation could be sent were solicited. Approximately three weeks following the initial mailing, a reminder was sent to those who had not yet responded.

Ethical considerations for this population-based study necessitated the identification of potential participants and their initial contact to originate from the provincial Vital Statistics office as a means to ensure confidentiality and privacy. Only bereaved family members who agreed to be further contacted were approached by the study team.

### Challenges and resolution strategies

A number of challenges were encountered during the initial months of this project, the majority of which had the potential to exert a substantial impact on the overall response rate and subsequent number of completed surveys. Some of the challenges were amenable to change while others were not in the control of the study team.

#### Indirect contact

Several challenges were encountered with the ethics board requirement of a third party to be responsible for identifying and contacting potential participants. The research team had no knowledge of who was invited to take part thereby maintaining confidentiality and privacy. Challenges with this process included unexpected delays in the distribution of study invitations due to third party workload and personnel changes, increased costs and the inability to estimate characteristics of non-respondents. However the major challenge was the necessity to place the onus on the bereaved family member to directly contact the study team themselves, which, for many, may have been perceived as an unnecessary burden adding to their grief.

Given the increasing concerns for personal privacy and confidentiality, challenges associated with the inability to directly contact potential participants and working with a third party will continue. Maintaining a positive working relationship that respects each other’s time, constraints and budgetary needs are of primary importance in order to continue this line of research.

#### Eligibility

In order to examine care provided at the end of life it is important that eligible decedents are accurately identified. Inclusion of people who died suddenly and did not receive EOLC services has the potential to impact results. In addition, including sudden deaths and informants who had no knowledge of the care provided as part of the denominator can result in the calculation of an artificially low response rate.

The ability to identify a death as being sudden or unexpected from the death certificate alone, beyond the external exclusions previously defined, are extremely limiting. As such, unless directly informed by a family member of the circumstances surrounding the death, sudden death survey information would be included. Compounding this problem is the possibility that many decedents who died suddenly were not provided EOLC. Because of this bereaved family members potentially saw no need to contact us for participation.

Although some bereaved family members contacted us directly to identify themselves as ineligible due to their lack of knowledge about their loved one’s EOLC or the decedent had experienced a sudden, unanticipated death, additional strategies to aid in the retrospective identification of eligible decedents and their family members were required. Conversations with the bereaved prompted the inclusion of two resolution strategies. The first involved asking the family member early in the survey administration if the decedent received care related to their health (with examples) in the last 30 days of life. The intent of this questioning was to help determine if the decedent’s health was failing during their last days and whether care, potentially related to end of life, was received. Although helpful in some instances, many decedents, whose death was considered sudden and unexpected by the family member, also received healthcare during this time. It was therefore difficult to determine whether this healthcare provided in the last 30 days could be considered EOLC. Even among decedents with advanced cancer, many bereaved family members did not believe the end of life was near or the bereaved themselves did not realize they did not have long to live. The second more successful strategy was the inclusion of a Frequently Asked Questions (FAQ) page with the invitation package. The intent of this FAQ page was to boost response rates by urging bereaved family members to inform us if the decedent had died suddenly and to encourage those who were unsure to contact us for more information. In addition to eligibility issues such as what to do if the death was sudden, if the decedent resided in a nursing home or whether Alzheimer disease was the cause of death, the FAQ page provided clear, simple answers to many general participant questions. This included answers to how their contact information was found and dealing with emotions during the survey interview. Following inclusion of the FAQ page with study invitations, the proportion of deaths identified as sudden by bereaved family members increased. At the same time, the number of telephone calls requesting clarification and additional study information decreased substantially.

#### Mailing procedure

##### Inaccuracies

A number of inaccuracies relating to the ‘informant’ contact information listed on the death certificate were drawn to our attention by the people receiving the invitations. At times, names and/or addresses were reported to be incorrect or misspelled. Of the incorrect addresses, some were returned but others were potentially lost if the bereaved family member was no longer at that address and the invitation disposed of. A small number of bereaved family members found an incorrect name to be distressing, particularly during this time of bereavement. In some situations, where family members or someone close to the decedent were not available, a funeral director, nursing home or healthcare provider was recorded as the death certificate informant, people who were ineligible to participate. Because we have no knowledge about these issues unless directly told by the person receiving the mailed invitation, the full extent and effect is unknown.

Unfortunately, errors in the name and address of the informant occur at the time of death certificate completion. Once informed of this problem, apologies were made and the correct information relayed to the third party for correction in the Vital Statistics database which appeased distressed family members.

##### Mail service delivery method

Study invitations were mailed using a recorded delivery priority mail service. Although very costly compared to regular mail, this service was selected in an effort to increase people’s attention to the receipt of the invitation package and to their perception of the study’s importance [24]. As well, all undelivered mail was assured to be returned to the sender which alerted the research team to a potential problem. However, priority mail service required someone to be at home at time of delivery. If not, a notice was left for the recipient to pick it up at the local post office which created an unanticipated burden for some, particularly the elderly who had difficulty travelling outside the home.

Due to the cost associated with the use of priority mail and the inconvenience it presented for some, a small nested study was initiated during the final wave of invitation package mailings. For the last wave, approximately half of the identified bereaved family members were contacted using the priority mail service whereas the second half received invitations through regular mail. As anticipated, the number of invitation packages using the regular service known not to be delivered was lower than those reported using priority mail. However, although the proportion of bereaved family members consenting to take part was somewhat (2.5%) higher among those who received their invitations by priority mail, the cost savings associated with delivery by regular mail was substantial (75% less).

#### Response rate and sample size

Our original intent was to send study invitations to a sample of family members or informants recorded on potentially eligible death certificates. However, following the first wave it was evident from the lower than anticipated response rate potentially due to ineligibility, that this strategy would not result in the targeted 1200 completed survey interviews. Our estimates were based on published response rates associated with after-death interviews studies [2,3], however, these teams were all able to contact family members directly and if contact could not be made, deemed ineligible. In this study the invited family member was asked to take an active step and contact the research team directly. At this difficult time it would be understandable for many to put the mailed invitation and information aside and not respond. Not having direct contact also resulted in little or no information being available about bereaved family members who declined participation or their eligibility. A lower than expected response rate is not only a reflection of the bereaved not able or willing to take part. It is also a function of the inclusion of ineligible family members of decedents who had died suddenly, those who lacked knowledge about the EOLC provided and others where the study invitation was unknowingly sent to those who had since died, been institutionalized or incorrectly identified. In a true response rate calculation, all are assumed eligible unless information is provided to indicate otherwise.

To help ensure the targeted number of completed surveys required to draw valid conclusions was obtained, all bereaved family members identified as potentially eligible were asked to participate beginning with the second wave. Because of the anticipated increase in the number of people willing to participate from each mailing, the total identified in each wave were randomly divided into two groups and mailed an invitation one month apart. This ‘split wave’ strategy worked very well and helped avoid long delays between the bereaved providing consent and contact by the survey interviewer. It also reduced research team and interviewer burden by controlling the number of people requiring contact at a single time point. Although costly and requiring additional funding sources, this strategy worked well and the number of completed surveys surpassed the target.

Additional strategies aimed to increase participation and to raise awareness of the project and EOLC issues in general included multiple interviews on provincial radio with the Principal Investigator and presentations to provincial palliative care program directors.

## Discussion

The administration of Canada’s first population-based mortality follow-back survey which gathered information about the experience of care during the end of life was successfully completed on time and surpassed the targeted number of survey interviews completed. However, during this process several challenges, some anticipated and others not, emerged. For the most part resolution strategies to help alleviate them proved beneficial. Nevertheless, in some instances it was not possible for the research team to affect change. These issues centered round the inability to directly contact potential participants, the identification of bereaved family members associated with an ineligible sudden death and inaccuracies in the informant field of the death certificate. Understanding these challenges and planning for them in the development stages of the research is recommended.

The importance of protecting personal privacy and confidentiality must be stressed. Debate about this issue has become prominent in many countries. Health information security and privacy acts are being developed or updated to protect personal information [25-28]. At the same time, it is necessary to acknowledge the benefits of health information use with respect to improvements in quality, safety and efficiency [26,28]. The inability to directly contact potential participants is an increasing reality. Limitations associated with this process are best acknowledged and addressed during study development including strategies to aid response. Efforts to ensure a positive, mutually respectful collaboration with parties aiding participant contact will help this form of research to move forward successfully.

Although many challenges could not be fully resolved, such as death certificate informant field inaccuracies and ineligible death identification, several resolution strategies employed were beneficial in aiding the research process. Of particular note was the inclusion of a FAQ page to answer questions that potential participants may have to encourage response, the use of a ‘split wave’ strategy to mail study invitations and the consideration of regular mail versus priority mail delivery.

The FAQ page proved to be a valuable tool to provide potential participants with the information they needed to make a decision about participation. Although much of the information could be found in the documentation required by the research ethics board, the question and answer format and language used in the FAQs was less formal, easy to follow, specific to this study and to the point. It also provided a vehicle for the research team to encourage bereaved family members to contact us directly with questions. Mailing of study invitations using a ‘split wave’ strategy over the two year data collection period successfully alleviated the challenge of all responses being received within the same time frame. As such, survey interviewers were able to contact consenting family members in a timely manner steadily throughout the collection period while at the same time, remaining sensitive to seasonal holidays and occasions.

Finding little difference in the response rate between the two mailing methods (regular versus recorded delivery priority mail service) used in the final wave was not expected. These results suggest no substantial benefits were associated with the use of priority mail service as a means of drawing attention to the study and encouragement of participation among bereaved family members. The use of a priority mail service proved to be inconvenient for some and substantially more costly than the regular mail. It will be important in future studies to consider this cost benefit ratio associated with modes of mail service.

In this article we focused on the challenges encountered during the process of implementing the study design. We would, however, be remiss not to acknowledge the potential burden participation in this study may have had on family members during their time of grief. Nevertheless it is also important to report that the vast majority of people who contacted us about participation were very positive about our objective to examine the experience of EOLC in our province from their perspective. Most explicitly expressed their appreciation for having the opportunity to talk about their loved one’s care and to potentially help in shaping future EOLC improvements. In addition, providers of EOLC were also keenly interested and supportive of this study’s results which provide a population-based perception of the experience of EOLC tapping many issues where little or no population estimates have been available [29].

In summary, although administration of a population-based mortality follow-back survey assessing the experiences of care during the end of life from the bereaved family members’ perspective has become increasingly challenging, due in large part to ethical and privacy concerns, this form of research can be successfully implemented and result in a wealth of information previously unavailable at a population level. Information gathered from the bereaved will provide a better understanding of the experience of EOLC which in turn can be used to promote positive change and better services for the dying and their families.

## Abbreviations

EOLC: End of life care.

## Competing interests

The authors declare that they have no competing interests.

## Authors’ contributions

BL participated in the conceptualization and design of the study, acted as coordinator, aided in the statistical analyses, drafted and finalized the manuscript. KV aided in the coordination of data collection, performed statistical analyses and helped to draft and finalize the manuscript. FB conceived the study, participated in the design and coordination and helped to finalize the manuscript. All authors read and approved the final manuscript.

## Pre-publication history

The pre-publication history for this paper can be accessed here:

http://www.biomedcentral.com/1472-684X/12/28/prepub
